# Process evaluation of the implementation of a decision support system to prevent and treat disease-related malnutrition in a hospital setting

**DOI:** 10.1186/s12913-021-06236-3

**Published:** 2021-03-25

**Authors:** Mari Mohn Paulsen, Cecilie Varsi, Lene Frost Andersen

**Affiliations:** 1 Department of Nutrition, Institute of Basic Medical Sciences, University of Oslo, box 1110 Blindern, 0317 Oslo, Norway; 2 National Advisory Unit on Disease-related Undernutrition, Division of Cancer Medicine, Oslo University Hospital, Rikshospitalet, Sognsvannsveien 20, 0372 Oslo, Norway; 3 Center for Digital Health Research, Oslo University Hospital, Division of Medicine, Aker hospital, box 4959 Nydalen, 0424 Oslo, Norway

**Keywords:** Process evaluation, Malnutrition, RE-AIM, effectiveness-implementation hybrid design, Decision support system, eHealth

## Abstract

**Background:**

Malnutrition is present in 30% of hospitalized patients and has adverse outcomes for the patient and the healthcare system. The current practice for nutritional care is associated with many barriers. The MyFood decision support system was developed to prevent and treat malnutrition.

**Methods:**

This paper reports on a process evaluation that was completed within an effectiveness trial. MyFood is a digital tool with an interface consisting of an app and a website. MyFood includes functions to record and evaluate dietary intake. It also provides reports to nurses, including tailored recommendations for nutritional treatment. We used an effectiveness-implementation hybrid design in a randomized controlled trial. The RE-AIM (Reach, Efficiency, Adoption, Implementation, Maintenance) framework was used to perform a process evaluation alongside the randomized controlled trial, using a combination of quantitative and qualitative methods. An implementation plan, including implementation strategies, was developed to plan and guide the study.

**Results:**

*Reach:* In total, 88% of eligible patients consented to participate (*n* = 100). *Adoption*: Approximately 75% of the nurses signed up to use MyFood and 50% used the reports. *Implementation*: MyFood empowered the patients in their nutritional situation and acted as a motivation to eat to reach their nutritional target. The compliance of using MyFood was higher among the patients than the nurses. A barrier for use of MyFood among the nurses was different digital systems which were not integrated and the log-in procedure to the MyFood website. Despite limited use by some nurses, the majority of the nurses claimed that MyFood was useful, better than the current practice, and should be implemented in the healthcare system.

**Conclusions:**

This study used a process evaluation to interpret the results of a randomized controlled trial more in-depth. The patients were highly compliant, however, the compliance was lower among the nurses. MyFood empowered the patients in their nutritional situation, the usability was considered as high, and the experiences and attitudes towards MyFood were primarily positive. Focus on strategies to improve the nurses’ compliance may in the future improve the MyFood system’s potential.

**Trial registration:**

The trial was registered in ClinicalTrials.gov 26/01/2018 (NCT03412695).

**Supplementary Information:**

The online version contains supplementary material available at 10.1186/s12913-021-06236-3.

## Introduction

Malnutrition due to disease is a large challenge in hospitals and about 30% of patients are malnourished or at risk of malnutrition [1–3]. Malnutrition is associated with increased morbidity and shorter survival, a longer length of hospital stay and increased hospital costs [1, 4, 5].

In response to the apparent lack of an effective system to monitor patients’ nutrition in the healthcare system, the MyFood decision support system was developed to aid in the prevention and treatment of disease-related malnutrition [6]. MyFood is a digital tool which includes functions for dietary recording, automatic evaluation of food intake compared to individual patient’s requirements and reports to nurses including tailored recommendations for nutritional treatment and a nutrition care plan for each patient [6, 7]. The effects of using MyFood has been studied in a randomized controlled trial [7]. The results showed that MyFood reduced the proportion of malnourished patients at hospital discharge and improved the nutritional treatment and documentation compared to the control group. However, no effect was seen on the primary outcome; weight change during the hospital stay [7].

A randomized controlled trial provides data on the effect of an intervention but offers limited insight into how the intervention worked, as well as how the intervention was affected by different barriers and facilitators and how they influenced the implementation [8]. Performing a process evaluation alongside a randomized controlled trial can, therefore, be useful to explore aspects of the implementation process [9]. Process evaluations aim to understand how complex interventions, such as MyFood, create change by evaluating implementation, mechanisms of impact, and the surrounding context when delivering the intervention [10].

Process evaluations can benefit from using a theoretical framework [10]. The Reach, Effectiveness, Adoption, Implementation, and Maintenance (RE-AIM) framework [11] presents the potential impact in terms of an intervention’s reach (proportion of the target population that participated in the intervention), effectiveness (patient outcomes), adoption (proportion of eligible settings that adopted the intervention), implementation (extent to which the intervention was implemented as intended) and maintenance (extent to which the intervention was maintained over time) [11]. These dimensions focus on the importance of measuring not only a traditional clinical outcome (i.e. effectiveness) but also implementation outcomes that are less frequently assessed [11]. Several studies have benefited from using the RE-AIM framework to perform a process evaluation alongside a randomized controlled trial [12–14].

To increase the opportunity for successful implementation, it is recommended to create an implementation plan which includes information about the specific actions that will be conducted in the implementation process, including implementation strategies, information about who will be responsible, the timeline and the expected outcomes [15].

The present study aimed to perform a process evaluation alongside the MyFood randomized controlled trial to evaluate the implementation plan and to understand implementation aspects to be able to prepare a scale-up of MyFood.

## Methods

### Design of the process evaluation study

This study reports on a process evaluation that was completed within an effectiveness trial by using an effectiveness-implementation hybrid design [16]. An effectiveness-implementation hybrid design promotes an examination of both effectiveness and implementation outcomes within the MyFood randomized controlled trial, as described by Landes and colleagues [17]. A hybrid type 1 design was applied, where the primary outcome constituted the effect of the MyFood randomized controlled trial [7] while performing a process evaluation to explore the implementation of the MyFood decision support system among the patients, the nurses and in the hospital setting.

### The MyFood decision support system

**Fig. 1:**
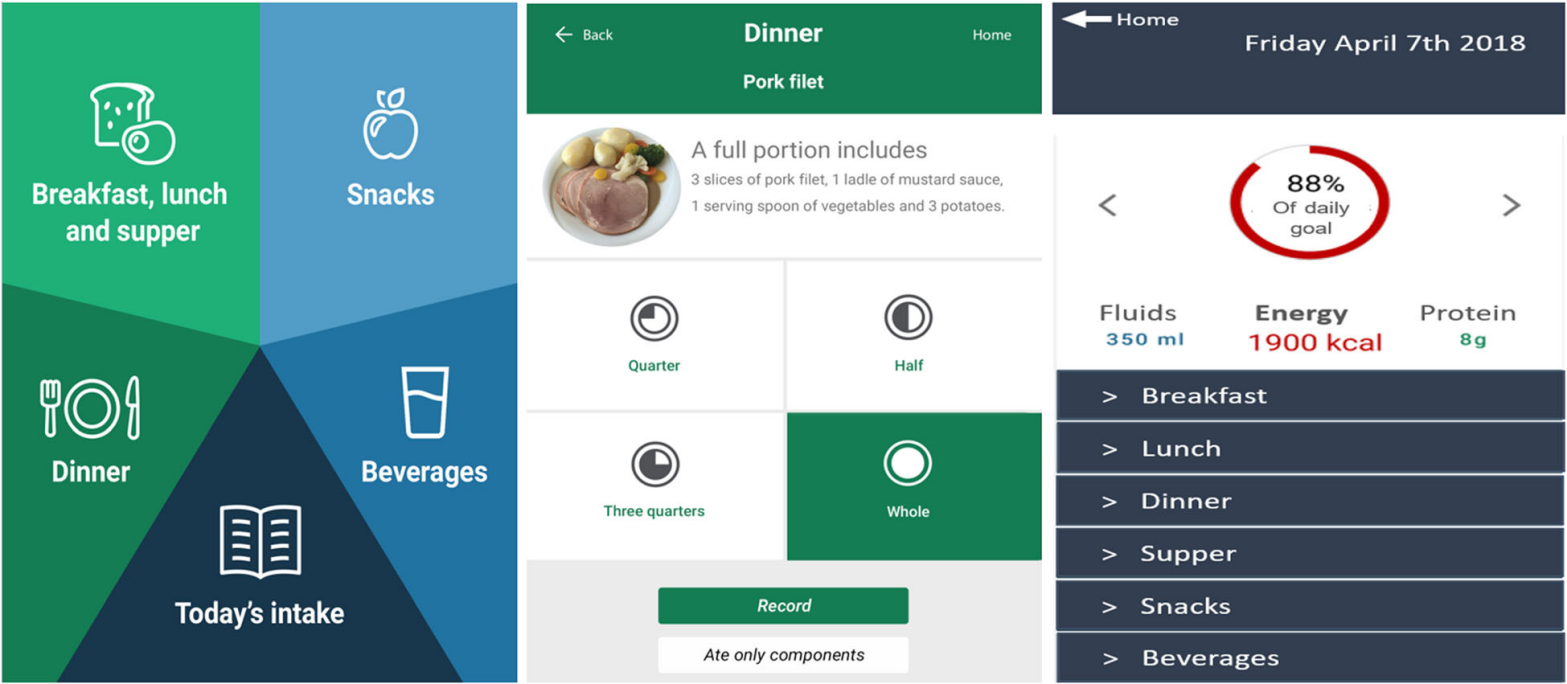
Dietary recording and evaluation in MyFood

MyFood is a decision support system designed to prevent and treat disease-related malnutrition. MyFood includes functions to record and evaluate patients’ nutrition and a report to healthcare professionals, including tailored recommendations for nutritional support and a template of an individualized nutrition care plan. The user interface of MyFood consists of an app including the dietary recording and evaluation functions and a website including the report function, as earlier described in detail [6, 7]. MyFood was used both by patients (the app) and nurses (the app and the website) in a hospital setting. To gain access to the MyFood website, the nurses needed to use a common log-in solution for public services in Norway [18]. Figure 1 illustrates the dietary recording and evaluation functions in MyFood.

### Setting and participants

The study was performed at a hematological department in a large university hospital in Norway. In total, 100 patients participated in the randomized controlled trial and 49 of these were enrolled in the intervention group and used MyFood during their hospital stay, as earlier described [7]. Approximately 120 nurses were employed at the hospital department and involved in the study.

### The implementation, as outlined in an implementation plan

A plan to guide the implementation of MyFood into the hospital department was created, as shown in Table 1. The plan included several implementation strategies [19], i.e. methods and techniques used when implementing MyFood to the hospital department. In this study, implementation strategies from the Expert Recommendations for Implementing Change (ERIC) project, which includes a compilation of 73 implementation strategies [20, 21], were used. The ERIC taxonomy was used to classify the strategies used.

**Table 1:** Implementation strategies included in the implementation plan for the MyFood decision support system

Phase	Description	Strategy (ERIC project)	Operationalization of implementation strategies in the present study
**1 Jan-Feb 2018**	Assess current situation and plan the implementation	**Use evaluative and iterative strategies** • Assess for readiness and identify barriers and facilitators. • Develop a formal implementation blueprint.	• Focus groups and interviews with healthcare professionals. • Planning the implementation process by making an implementation plan.
**2 March-July 2018**	Preparation and training	**Adapt and tailor to context** • Tailor strategies. • Promote adaptability. **Train and educate stakeholders** • Conduct educational meetings. • Develop educational materials. • Distritbute educational materials. **Develop stakeholder interrelationships** • Recruit, designate and train for leadership. • Identify and prepare champions. • Use an implementation advisor.	• Tailor strategies to context based on results from phase 1. • Provide support for nurses’ access to the MyFood website. • Education to nurses at the department. • Written material to nurses and patients about MyFood and the randomized controlled trial. • Distribute materials by e-mail and brochures. • Include leaders in planning of the trial. • Provide responsibility to leaders for distribution of information and involvement in patient recruitment and follow-up of the trial. • Employ reasearch nurse from the department as a super user and “champion”. • The second author (CV) filled this role.
**3 Aug 2018- May 2019**	During the randomized controlled trial	**Develop stakeholder interrelationships** • Organize clinician implementation team meetings. • Identify early adoptors. **Train and educate stakeholders** • Conduct ongoing training. • Provide ongoing consultation. **Support clinicians** • Remind clinicians. **Provide interactive assistance** • Provide local technical assistance. • Provide clinical supervision. **Engage consumers** • Involve patients/consumers and family members. • Intervene with patients/consumers to enhance uptake and adherence. • Prepare patients/consumers to be active participants.	• Discussion of different aspects, challenges etc. • Encourage early adoptors to promote MyFood and positively influence others. • Continuos training in group or one-to-one. • Availability of project workers every weekday. A project phone available all times. • Inform and remind nurses to follow-up the MyFood reports. • Support nurses with access and log-in. • Guidance in use of MyFood for patients and nurses when needed. • Train the research nurse to teach and support the other nurses at the department. • Encourage patients to use the MyFood app daily. Next-of-kin are welcome to contribute. • Provide support to patients. • Provide verbal and written information. Patients will be encouraged to ask if they have questions.

The first phase of the implementation plan involved the assessment of potential barriers and facilitators of using MyFood in the hospital department, using the Consolidated Framework for Implementation Research (CFIR) [22]. This phase was conducted before MyFood was implemented to the hospital department and the results of this assessment are described elsewhere [23]. Further, implementation strategies were selected based on the findings in the first phase, to address the identified barriers and facilitators before the implementation of MyFood to the hospital department.

The second phase of the implementation plan involved preparations at the hospital department and the training of healthcare professionals. The third phase included a plan for implementing MyFood during the randomized controlled trial. The operationalization for performing the various ERIC strategies in the implementation plan is described in Table 1.

### The RE-AIM framework and data collection procedures

The RE-AIM framework [11] was used to guide the process evaluation. Table 2 shows the five RE-AIM dimensions: reach, effectiveness, adoption, implementation and maintenance, and how they were assessed during the process evaluation in the randomized controlled trial using both quantitative and qualitative methods.

**Table 2:** Overview of the operationalized RE-AIM dimensions and how they were covered in the process evaluation

RE-AIM dimension	Description	Data collection	When
Reach	Proportion of patients who participated in the trial and reasons for decline	Register the number of patients who consent to participate in the randomized controlled trial (n = 100). Register age, gender and reason for patient decline in the trial (*n* = 20)	Within 48 h after hospital admission
Effectiveness	MyFood’s ability to prevent and treat malnutrition	Reported elsewhere [7]	During the patients’ hospital stay
Adoption	The proportion of nurses who used the MyFood system	Technical log data (*n* = 63). Questionnaires to nurses (n = 68)	Log data: During the trial. Questionnaires: At the end of the trial
Implementation	Fidelity to the MyFood system. Attitudes and experiences with the use. Usability of MyFood.	Questionnaire to nurses (n = 68) and patients (n = 40). Focus groups with nurses (3 groups, *n* = 14 in total). Individual interviews with patients (*n* = 12)	At the end of the data collection period for the nurses. At the end of the hospital stay for the patients
Maintenance	The extent to which the intervention was sustained over time	The data collection was performed during a limited period; hence we did not have the opportunity to study maintenance over a longer period	Not measured

Several procedures were used to collect data:

- **Questionnaires**

Questionnaires were distributed to both patients and nurses, to assess the RE-AIM dimensions *adoption* and *implementation*.

A short study-specific paper-based questionnaire including five questions about the usability of the MyFood app and awareness of own nutritional situation was collected from the patients in the intervention group who used MyFood during their hospital stay. The patients completed the questionnaire close to hospital discharge.

A more comprehensive web-based questionnaire was distributed to the nurses towards the end of the data collection period in the randomized controlled trial. The form included a total of 16 questions about the nurses (age, sex, type of position, and work experience), use of the MyFood app, use of the MyFood website, experiences with use of and attitudes towards MyFood.

- **Technical log-data**

Data on the use of the MyFood app by patients and nurses were continually sent to the secure data storage platform “Services for sensitive data” (TSD) [24], as described earlier [7], and hence we could track the recording of food intake in MyFood, providing information to the RE-AIM *implementation* dimension. We were also able to retrieve information about the nurses’ use of the MyFood website, by studying the technical logs of accessing the MyFood website, providing information to the RE-AIM *adoption* dimension.

- **Focus group discussions with nurses and individual interviews with patients**

Qualitative focus group discussions with nurses and individual interviews with patients provided information to the RE-AIM *implementation* dimension.

The interview guides included the following topics: Use of MyFood, usability, training, experiences, attitudes, usefulness, communication and culture (Supplementary files 1 and 2). The same topics were included in both staff focus groups and patient interviews, but the questions were tailored to each group of informants.

Three focus groups were conducted with nurses in February 2019 at a meeting room at the hospital department. The nurses were purposively selected for the focus groups by the nurse manager at the hospital department, based on availability and shifts the days the focus group discussions were conducted. A secretary assisted the first author (MMP) and each focus group included 4–5 nurses and lasted from 46 to 48 min. The first focus group served as a pilot to test the formulation of questions and the interview guide. After the focus group, the nurses were asked about the relevance of the questions and the setting of the focus groups. No changes of importance were made to the interview guide after the pilot focus group and the data from this group were therefore included in the analysis.

Semi-structured individual patient interviews were conducted by the first author (MMP) among the first 12 patients who were discharged, after the first initial month of data collection in the randomized controlled trial. The interviews were conducted at the patient’s room at the hospital department and lasted from 5 to 15 min, 10 min on average.

The focus groups and interviews were audiotaped with a digital voice recorder (Olympus WS-853) and transcribed verbatim, using the software f4transkript (Marburg). Notes were also taken by the secretary during the focus group discussions and the first author took notes immediately after the individual interviews.

### Data analysis

The data from the patient questionnaires were analyzed using the IBM SPSS statistical software version 24. The web-based questionnaire to the nurses used a webform [25] which generated a report summarizing the nurses’ responses. The technical log-data from MyFood were analysed in Microsoft Excel 2016 after they were retrieved from the TSD server.

The transcripts from the focus groups and interviews were analyzed using thematic analysis in a stepwise manner as described by Braun and Clarke [26]. The software NVivo version 12 (QSR International) was used to perform the analysis. First, the transcripts were read by the first author to get an overall overview of the material. Second, initial codes were created. Third, themes were established deductively for the RE-AIM dimension ‘Implementation’. More specifically, this included the use of MyFood, usability aspects, experiences and attitudes. In addition, a theme for implementation strategies was established. Fourth, the codes and themes were reviewed to establish the meaning and to interpret the results.

Trustworthiness in the analysis [27], including credibility, confirmability, dependability and transferability [28], was emphasized. This involved the inclusion of both nurses and patients in the interviews, and audio taping and transcribing the material verbatim. It also included analyzing the data systematically, involving all authors in the development of the interview guide, including the first (MMP) and the second (CV) authors in the development of the coding categories and the interpretation of the results.

### Ethical considerations

The study was performed following the Helsinki declaration and was approved by the Norwegian Committee for Medical Research Ethics (2016/1464) and the data protection authority at the hospital. Informed consent was collected from all participants. The study was registered at the National Institutes of Health Clinical Trials 26/01/2018 (www.clinicaltrials.gov/; Identifier: NCT03412695).

## Results

### Study participants

The patients in the intervention group who used MyFood during the randomized controlled trial were 50 years on average and 71% were men. The nurses at the hospital department were 34 years on average and 88% were women.

The 12 patients who participated in the individual interviews were 52 years on average (range 27–65) and 67% were men. The focus groups included 14 nurses in total, they were all female with a mean age of 32 years (range 23–49). The groups were composed of nurses with about the same level of work experience.

### Results from the process evaluation

The process evaluation assessed the implementation of MyFood during a randomized controlled trial through the aspects of the RE-AIM dimensions and the strategies in the implementation plan (Table 2).

#### Reach

*Reach* refers to the participation rate within the MyFood randomized controlled trial and characteristics of participants and nonparticipants [11]. In the MyFood randomized controlled trial [7], 120 patients were considered eligible and were requested to participate in the trial. Out of these, 100 patients (83%) consented to participate. Table 3 shows the characteristics of the patients who approved consent, compared to the patients who declined to participate.

**Table 3:** Characteristics of patients who approved consent and declined to participate in the randomized controlled trial

	Approved to participate (***n*** = 100)	Declined to participate (***n*** = 20)
Sex, male	60 (60%)	10 (50%)
Age (years)	51.9 (15)	48.5 (16)

Data are presented as the number of participants (%) or the mean (SD)

The patients who declined to participate in the trial were from 19 to 68 years, with a mean age of 49 years. The reasons for declining participation are shown in Table 4.

**Table 4:** Patients’ reasons for declining participation in the MyFood randomized controlled trial (*n* = 20)

Reason for declining to participate	n (%)^a^
Too many worries with the disease. Becomes too much to participate in a trial	8 (40)
Cannot stand the focus on food and recording	4 (20)
Do not want to participate	2 (10)
Already included in other trials	3 (15)
Unfamiliar with the use of apps and tablet computers	3 (15)
Not confident in the Norwegian language	1 (5)
Previous experience with eating disorders	1 (5)

^a^Some patients are included in several categories; therefore, the total percentage equals more than 100

Some patients (n = 5) were not asked to participate in the trial because the responsible nurse considered the patient as unable to attend because of difficulty communicating with the hospital staff in general (*n* = 3) or the presence of sepsis and a poor general condition (*n* = 2).

#### Adoption

Technical log-data indicated that 75% of eligible nurses signed up to use MyFood. The web-based questionnaires revealed that the most important reasons for not signing up were skepticism towards the log-in solution (i.e. personal login through a secure log-in solution for public services in Norway, bank-ID [18]) (*n* = 7) and lack of time (*n* = 5). Other reasons were ‘did not know how’ (*n* = 1), ‘not interested’ (n = 1) and ‘did not want to’ (n = 1).

Table 5 shows the results from the web-based questionnaire about the nurses’ use of MyFood. Three-quarters of the nurses reported to use the MyFood app and about 60% used the report function in the MyFood website. About half of the nurses used the information provided in the report function to document the patient’s nutritional intake in the electronic patient record, and about one third used MyFood to create nutrition care plans. About half of the nurses reported using MyFood as a basis for dialogue with the patient about their nutritional situation or to implement nutritional treatment. The majority of the patients did, however, not experience the nurses using MyFood to have a dialogue about their nutritional intake and situation.

**Table 5:** The nurses’ use of MyFood, *n* = 68^a^, based on data from the web-based questionnaire

	n (%)
Used the MyFood app	51 (75)
Used the MyFood website (report function)	43 (63)
Used website to document nutrition in the electronic patient record	37 (54)
Used website to create nutrition care plans	24 (35)
Used website for dialogue with the patient or to implement nutritional treatment	36 (53)

^a^The web-based questionnaire distributed to the nurses by the end of the data collection period received a response rate of 69%

#### Implementation

##### Implementation of MyFood among the patients

As previously reported [7], patient compliance of using MyFood was high. The compliance, defined as the number of days recorded in the MyFood app divided by the patient length of hospital stay, was 93% ranging from 73 to 100%.

Patients’ perceived usability of MyFood is shown in Table 6. All patients reported that MyFood was easy to use, although some had comments on how the app could improve to be even more user-friendly. The majority of the patients were able to find the food and beverage items they wanted to record in the MyFood app and managed to record the correct amount. Few patients experienced a need to obtain new knowledge to be able to use MyFood (Table 6). One of the patients said:

**Table 6:** Perceived usability^a^ of MyFood among the patients (*n* = 40)

	Totally disagree	Disagree	Neutral	Agree	Totally agree
	n (%)^b^				
I found MyFood easy to use	0 (0)	0 (0)	0 (0)	10 (25)	30 (75)
I found the food and beverages I wanted to record	0 (0)	0 (0)	5 (13)	21 (53)	14 (35)
I managed to record the correct amount	0 (0)	0 (0)	4 (10)	12 (30)	24 (60)
I needed to obtain new knowledge to use MyFood	33 (83)	4 (10)	2 (5)	0 (0)	1 (3)

^a^Data from the paper-based questionnaire

^b^40 of 49 patients in the MyFood intervention group completed the usability form

“*I have never used an app before, so for me, this was something new. It was a bit challenging and exciting. I was impressed that I - a beginner, managed to do it. I was very satisfied*.*”* [Patient]

The patients reported that MyFood motivated them to eat more to reach their nutritional targets:

“*When the appetite is at the bottom, I think you manage to eat more than you would have done without MyFood. It provides an overview, you can see if you reach your targets, right.*” [Patient]

However, not all patients were motivated.

“*I think it differs from patient to patient. For some, it has been ‘gold’, because they have been able to record and obtained a complete overview of their nutrition. It’s much better than that paper-form. For a few, it has been more strenuous*”. [Nurse]

Based on the questionnaire provided to the patients at the end of their hospital stay, 88% reported becoming more aware of their nutritional situation after use of MyFood. When interviewing the patients, many said that the use of MyFood had made them more aware of their nutritional requirements:

‘*It [the MyFood app] has given me a better overview. … I saw what types of food that contributed a lot and which types contributed less. That had a large impact. I understood how different types of food contributed*. … *In periods where I needed to increase my body weight, the app was perfect for me.*’ [Patient]

The value of increased control of the nutritional situation for the patients themselves was emphasized:

*“The app was continuously updated with my calorie intake. I did not have to write down on paper and use Google to find the energy content. I had control*”. [Patient]

Several patients talked about MyFood and their nutritional situation with their next-of-kin and/or friends:

“*I think I have shown it [MyFood] to everyone - to my children and wife, and my buddies who visited and are interested in nutrition and that kind of stuff, so I have shown it to the people who have been here. It is probably because I find it exciting.*” [Patient]

##### Implementation of MyFood among the nurses

The implementation strategy of *identifying and preparing champions* was appreciated by the nurses:

“*It was brilliant to have two nurses working here involved. It was easy to ask because we know them so well. … They have been very accessible and provided great support.”* [Nurse]

Before being able to record dietary intake, the patient had to be registered in the MyFood app with information about the patients’ body weight, height, age and nutrition-related symptoms. When developing the MyFood system and planning the study, this was supposed to be the nurses’ task and the nurses received training and written information. However, the nurses reported that they perceived it more appropriate for the patient to perform this task themselves:

“*The MyFood app empowers the patient for their nutritional situation and they can just as well be responsible for registering their body weight etc … They are the one knowing their own symptoms best. I have not seen any problems with the patients performing this task themselves*”. [Nurse]

In general, there seemed to be a perception among the nurses that the patients were the ones who used MyFood the most:

“*I did not use it [MyFood] much. But I experience that it has a large value for the patient. They find it easy to use and they obtain a good overview, so it was limited how much I needed to contribute. They have registered information and arranged the use pretty much themselves. However, after I overcame the obstacle of logging into the report function and obtain recommendations I discovered that it was very useful.*” [Nurse]

The hospital department currently deviated from the recommended practice for prevention and treatment of disease-related malnutrition, e.g. related to recording and evaluating nutritional consumption [23], and therefore the use of MyFood represented a new, and additional task to perform:

“*MyFood is an extremely useful tool. It would be of great value for patients and healthcare professionals if MyFood was permanently implemented. However, the use of MyFood by the nurses is probably perceived as more time consuming, because regular nutritional routines are not performed properly. After all, if you perform the routines [guidelines for the prevention and treatment of malnutrition] properly – they are very time-consuming and cumbersome*.” [Nurse]

During the focus groups, several nurses claimed that the MyFood report function with recommendations for nutritional treatment was very useful and used it a lot:

“*I think it is brilliant. It becomes a supervisor and at the same time, you can combine the tailored recommendations with your suggestions*.” [Nurse]

At the same time, other nurses claimed that this function might be too much in an already busy schedule.

“*It may be too much because it requires that you have to sit down, read and make considerations. But, at the same time, if you have a rehabilitation case like we sometimes have, I think this information will be very useful. If you have time to sit down and focus on nutrition … But I have to admit, I have not used this function a lot*.” [Nurse]

A barrier for use of MyFood was different digital systems at the hospital department which were not integrated:

*“I have used MyFood a lot after I first started to use it, but at first it was like “ah, yet another computer system …*” *, that’s how it felt. We already have several platforms we have to log into … It becomes much hassle.”* [Nurse]

##### Nurses’ attitudes and experiences with the use of MyFood

**Fig. 2:**
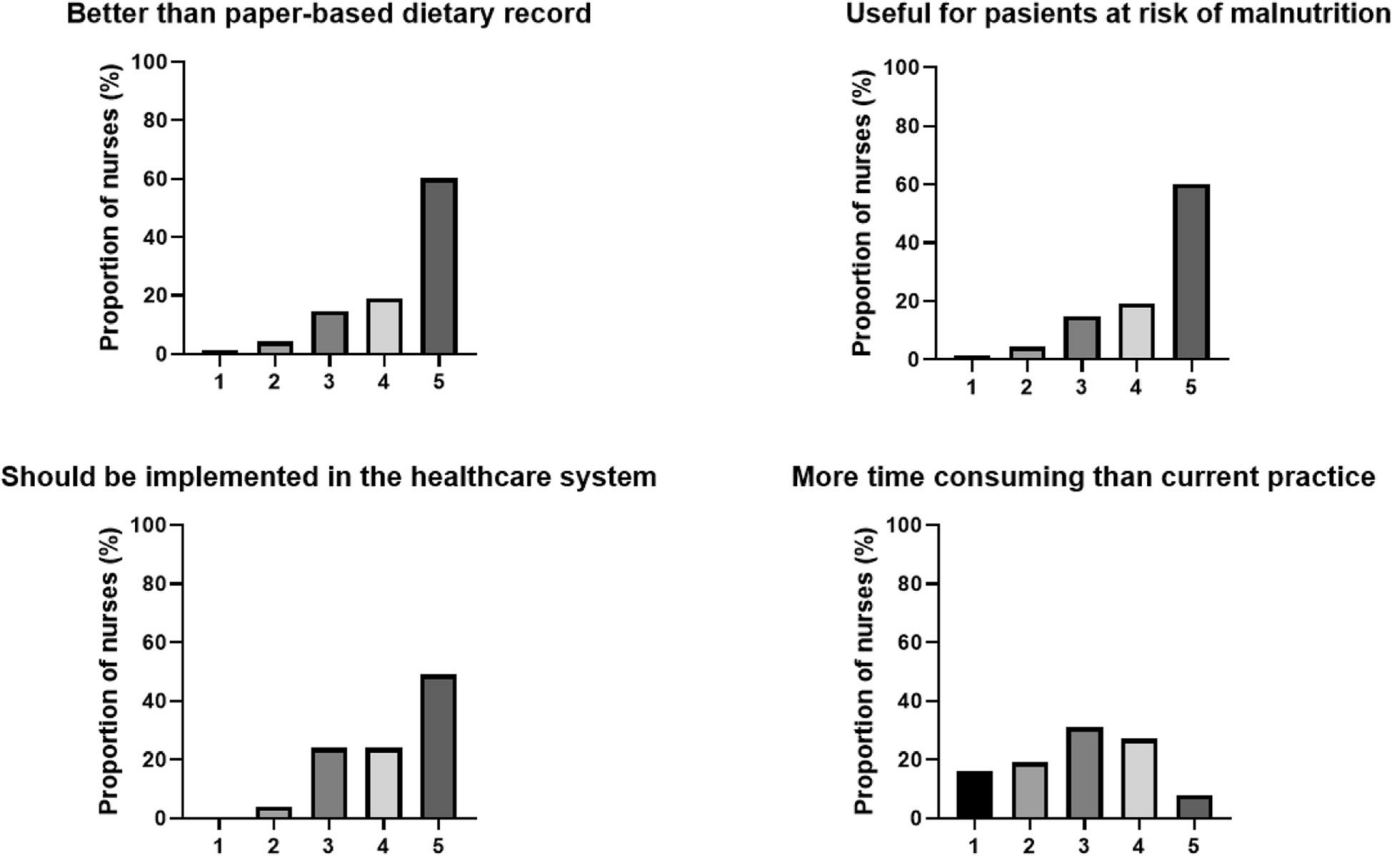
Attitudes and experiences among nurses (*n* = 68) with the MyFood system, based on the Likert 5-point scale ranging from 1: ‘Totally disagree’ to 5: ‘Totally agree’

Figure 2 illustrates the results from the web-based questionnaires regarding attitudes and experiences with the use of MyFood among the nurses. In total, 81% of the nurses reported that the use of MyFood for dietary recording was better than the current practices with paper-based recording, 79% reported that MyFood was useful to monitor patients at risk of malnutrition, and 72% reported that MyFood should be implemented in the Norwegian healthcare system.

The nurses experienced that use of MyFood motivated the patients:

“*A lot of the patients used MyFood as motivation. … A majority does not have a lot of nutrition knowledge. They do not know what to eat to... so I think many patients feel like this: “I have control now. I only need to eat 20% more to reach my target”. They use it as a measurement scale to go to the kitchen to get some food. I think a lot of the patients have done that*.” [Nurse]

An impression of increased patient knowledge about nutritional requirements among the patients was a consistent finding among the nurses:

“*… I think they learn a lot by using MyFood. It’s an advantage that they understand how much they have to eat to cover their requirements. They don’t use that form [paper-based dietary recording form] lying on the nightstand, but they use MyFood because they can fix it themselves*”. [Nurse]

The value of increased control of the nutritional situation for the patients themselves was emphasized:

“*We mess about so much, you know: Have you been to the toilet? Have you been in the shower? Have you taken your temperature? Have you been on the scale? For MyFood it became like, “I have control of this [the nutrition], this is my thing [MyFood]. I think they [the patients] experience that they have something they have control of themselves and that we should not mess about. It [MyFood] reports automatically, so we do not have to constantly go into their room and mess about everything*”. [Nurse]

Several nurses reported that the patients became proud when they managed to reach their nutritional target and wanted to show the nurse what they had obtained:

“*I experience that some patients become a little bit proud. “Look, today I have reached 85% of my need.” … It gives them a sense of achievement. They have something to work towards and maintain the control themselves*.” [Nurse]

## Discussion

This study reported on a process evaluation that was completed within an effectiveness trial. The MyFood system reached 83% of eligible patients and no large differences were seen between patients who approved or declined to participate. The MyFood decision support system was adopted by 75% of the nurses working at the hospital department. The compliance of using MyFood was higher among the patients than the nurses. The majority of both patients and nurses perceived MyFood as easy to use, but the MyFood app was used more and perceived as easier to use than the report function on the MyFood website. Use of MyFood during the hospital stay empowered the patients by increasing their motivation to eat to reach their nutritional targets, increasing their knowledge/awareness about nutrition and their sensation of control. A barrier for use of MyFood among the nurses was different digital systems at the hospital department which were not integrated and the log-in procedure to the MyFood website. Despite limited use by some nurses, the majority of the nurses claimed that MyFood was useful, better than the current practice with paper-based dietary recording forms, and should be implemented in the Norwegian healthcare system. To the best of our knowledge, no published process evaluation studies examining the implementation of a decision support system for nutritional support in hospitals exist.

### Reach of the MyFood system

The MyFood system reached the majority of eligible patients and no particular pattern was observed concerning differences between patients who wanted to participate and patients who declined. The most common reason for declining was too many worries about the disease. A patient who suffers from severe disease, may not have the surplus to be involved in clinical trials. Only three patients did not want to participate due to unfamiliarity with apps and tablet computers. Older age has been described as a potential barrier to the use of new technology [29], whereas other studies have demonstrated that elderly people often are positive about using tablet computers and eager to learn [30, 31]. The patients who participated in the MyFood intervention study were relatively young compared to the general hospital population. Therefore, we do not know if an older age is a barrier for use of MyFood. A concern is that some patients were not asked to participate, due to considerations of the responsible nurse. Future strategies should increase focus on how to ensure that all patients are provided with the same possibilities for using MyFood.

### Patient empowerment

Empowerment can be defined as the process of helping people to assert control of the factors which affect their health [32] or as a continuous process through which patients work in partnership with their healthcare system [33]. In the present study, patients reported feeling empowered in their nutritional situation when they used MyFood during their hospital stay. The MyFood app seemed to provide value directly to the patient by being a tool that made the patient more able to take control of their nutritional situation and make them keep track of intake compared to requirements. This finding corresponds with a recent integrative review which indicated that patients experience increased empowerment through technology-enabled care solutions [34]. A recent Australian study about hospital patients’ perceptions of using a technology-based nutrition intervention also found that recording food intake and viewing their goals made them more aware of their nutrition [35]. The involvement of the patient in the use of decision support systems is shown to be more effective than only involving the health-care professional [36]. Roshanov et al. [36] argued that this probably is due to the increased empowerment of the patients when they become actively involved in their care. Mahmood et al. [37] found that patients using mHealth applications to track progress on a health-related goal performed health-related decisions and engaged in health-related discussions with the healthcare providers to a larger extent than patients not using such mHealth applications.

A consistent finding in the present study was that the use of MyFood motivated the majority of the patients to reach their nutritional targets. This corresponds with results from a recent scoping review of the use of eHealth systems to patients with type 2 diabetes, where the eHealth systems contributed to increased motivation, awareness and change of habits to follow the allocated treatment [38]. The possibility to keep track of nutrition was also found to motivate the patients in a Danish qualitative study of hospitalized patients’ experiences of being undernourished [39] and in an Australian study describing patients’ perceptions [35] and hospital staff’s perceptions [40] of an electronic program to engage patients in nutrition care bedside.

As previously reported [7], the MyFood intervention decreased the proportion of patients at risk of malnutrition at hospital discharge, compared to the control group. This may be due to the effect MyFood had on motivating the patients to eat to reach their nutritional targets and also the finding that MyFood made the patients more aware of their nutritional requirements. Increased empowerment among the patients may explain the high patient compliance. It might be beneficial to focus future implementation strategies on how MyFood may increase empowerment also among the nurses in their nutritional support.

### The use of implementation strategies

A realist systematic review identified that the most important strategies for implementation success were management support and engagement, internal and external facilitation, training, and audit and feedback [41]. In the present study, the *use of evaluative and iterative strategies* through the *assessment for readiness* and the *identification of facilitators and barriers* in advance and *development of stakeholder interrelationships* by *identifying and preparing champions* was useful to plan the trial. However, the implementation strategies were not tested systematically.

In the implementation plan, we aimed to include the leaders by the implementation strategy of *recruiting, designating and training for leadership*. Leaders were included in the planning of the trial and had the responsibility to distribute information about the need for all the nurses to sign up to get access to MyFood before the initiation of the trial. The leaders’ role in making the nurses sign up was important, however, we did not experience that the leaders took an active part and responsibility beyond this.

### Higher compliance among the patients than the nurses

We found that the patients were highly compliant with the use of MyFood, whereas the nurses were less compliant. The results indicated that the nurses were pleased with the patients taking control of MyFood themselves, doing the dietary recording and keeping track of their food intake. Some nurses even expressed a perception of this monitoring being the patient’s task. Few patients experienced that the nurses used MyFood to have a dialogue about their nutrition. According to Norwegian [42] and European [43] guidelines, nutritional care should be an interdisciplinary task. The Norwegian Patient’s Rights Act is following international human rights and states that all patients have a basic right to receive treatment and care according to their needs [44]. According to the Healthcare Personnel Act [45], the patient should receive an individual treatment plan, including nutrition. It should be emphasized to healthcare professionals that even though MyFood can empower the patients and make them more aware of own nutrition, the responsibility for appropriate nutritional documentation, monitoring and treatment still lays with the healthcare professionals and is not something the patient should have to deal with themselves.

### Barriers and facilitators to implementation

The most important barriers to use of MyFood among the nurses were the lack of automatic integration with the electronic patient record and the log-in procedure to the MyFood report function. Lack of integration with the electronic patient record was also identified as the main barrier for implementing an eHealth intervention for symptom assessment and communication into a clinical hospital setting in Norway [46]. Future development should look into possibilities for integration with the electronic patient record and explore alternative log-in procedures.

The use of nurse champions was an effective strategy and considered as an enabler for the use of MyFood. This corresponds to other findings of nurse champions promoting innovations in nutrition care [47, 48] and the implementation of eHealth [49]. Potential future implementation of the MyFood system in healthcare should consider increasing the use of champions to enable local implementation.

### Strengths and limitations

A strength of this study involved the creation of an implementation plan, including implementation strategies, based on the initial identification of perceived barriers and facilitators [23]. The implementation strategies were, however, not tested in a systematic manner in the present study.

Using an established framework to guide the process evaluation is considered an important strength to better understand, describe and identify factors that affected the implementation of the MyFood system. Although several approaches to evaluate the implementation of intervention exists, we considered the RE-AIM framework to be most suitable for the present study. Other frameworks, as the conceptual framework for implementation fidelity [50] and Proctor’s implementation outcomes [51] were also considered.

Another strength of the study is the inclusion of both patients and nurses to reveal different perspectives. The focus groups were composed of nurses with the same level of work experience to facilitate that everyone was heard and to enable the more inexperienced nurses to talk freely in a separate group that was not dominated by more experienced nurses. This worked out well and all participants were active and expressed their views and opinions.

A limitation is that this study was conducted at one hematological department at one hospital and the results may not be representative in other patient groups and for other healthcare settings. The nurses were purposively selected for the focus groups based on availability and shifts the days the focus group discussions were conducted. As a consequence, nurses primarily working night shifts or week-ends may not have been represented. Thus, this study does not necessarily provide knowledge about the implementation of MyFood during night shifts and in week-ends.

### Suggestions for future research

To be able to understand the generalizability of the results in this study, effects and implementation of MyFood should be explored in other patient groups and different healthcare settings. Future research should focus on strategies for how to increase compliance among healthcare professionals in the use of eHealth systems for nutritional support. Hospitals are described as challenging and complex environments and even when successfully implemented, interventions frequently stop being delivered after initial funding had ceased [52]. There is a large need for digital solutions for nutritional monitoring and support in the hospital setting and research on how to overcome barriers to the sustainability of such solutions is needed to be able to prepare scale-up.

## Conclusion

This study used a process evaluation to provide more in-depth understanding of the results of a randomized controlled trial to be able to prepare the scale-up implementation of the MyFood decision support system into a clinical hospital setting. The patients were highly compliant and used MyFood as intended, however, the use was lower among the nurses. The MyFood system empowered the patients in their nutritional situation, the usability of MyFood was considered to be high, and the experiences and attitudes towards MyFood were primarily positive. A barrier for use among the nurses was the log-in solution and lack of integration between different digital platforms at the hospital. Focus on strategies to improve the nurses’ use and follow-up may in the future improve the MyFood system’s potential.

## Supplementary Information

**Additional file 1: Supplementary file 1.** Interview guide nurses.

**Additional file 2: Supplementary file 2.** Interview guide patients.

## Data Availability

The datasets generated and/or analysed during the current study are not publicly available, but may be available from the corresponding author on a reasonable request.

## Abbreviations

CFIR: Consolidated Framework for Implementation Research
ERIC: The Expert Recommendations for Implementing Change project
RE-AIM: The Reach, Effectiveness, Adoption, Implementation, and Maintenance framework
TSD: Services for sensitive data [Tjenester for sensitive data]
