# Supplementary material for: Process evaluation of the implementation of a decision support system to prevent and treat disease-related malnutrition in a hospital setting
Source: BMC Health Serv Res. 2021 Mar 25;21:281. doi: 10.1186/s12913-021-06236-3 (PMC7995565; doi:10.1186/s12913-021-06236-3)
Supplement: Supplementary file 1 — Additional file 1: Supplementary file 1. Interview guide nurses. [file 12913_2021_6236_MOESM1_ESM.docx]

**Process evaluation of the implementation of a decision support system to prevent and treat disease-related malnutrition in a hospital setting**

Mari Mohn Paulsen^1,2^, Cecilie Varsi^3^, Lene Frost Andersen^1^

**Author affiliations:**

^1^Department of Nutrition, Institute of Basic Medical Sciences, University of Oslo, box 1110 Blindern, 0317 Oslo, Norway.

^2^National Advisory Unit on Disease-related Undernutrition, Division of Cancer Medicine, Oslo University Hospital, Rikshospitalet, Oslo, Norway. Sognsvannsveien 20, 0372 Oslo, Norway.

^3^Center for Digital Health Research, Oslo University Hospital, Division of Medicine, Aker hospital, box 4959 Nydalen, 0424 Oslo, Norway.

**Corresponding author**: Mari Mohn Paulsen

[m.m.paulsen@medisin.uio.no](mailto:m.m.paulsen@medisin.uio.no)

**Supplementary file 1. Interview guide nurses**

| **Introduction** | Purpose of the interview, practical information (audiotaping, confidentiality). |
| --- | --- |
| **Opening questions**  **About the study**  **Training/equipment**  **The use of MyFood**  **Experiences** | In general, what do you think about the MyFood tool?   - What is good? - What is challenging?   What kind of information did you receive about the MyFood tool and the study before start-up?  How was the training you received?   - What was useful? - Any information that was missing? - Anything that should have been available or facilitated for easier implementation of the tool in a hospital setting?   How did you use the MyFood app?   - Registration of the patient in the app - Registration of diet - Registration of TPN/EN - Easy/difficult to remember? - The need of support when using the app?   How did the patients use the app?   - Did the patients use the app? - Did the patients remember to record their nutritional intake? - What was the advantages with the patients reporting their intake in the app? - What was the challenges with the patients reporting their intake in the app?   How did you use the MyFood report function?   - Access? - Login? - How often did you collect patient reports? - Perceptions of the content? - What did you think about the suggestions for the nutritional measures? - Did you carry out any of the measures in practice? - Did you implement the draft for the nutritional plan? - What was good? - Was there anything missing? - Something that should have been different?   What do you think about the usability of MyFood?   - For the app? - For the report? |
| **Communication and culture** | To what extent did you talk to your patients who participated in the study about the app?  To what extent did you talk to other nurses about the MyFood tool?  To what extent did you talk to your manager about the MyFood tool?  What was communicated from the management about the use and follow-up of the MyFood tool? |
| **Participation in the study** | How did you experience to participate in this study/having a study conducted at your ward?   - Other ongoing studies at the same time? - How did that affect the focus of this study?   Is there anything that should have been done differently for better implementation of the system? |
| **Perceived utility** | Do you have any thoughts about the use of MyFood compared to other measures for nutritional follow-up?  What have you learned about nutrition, which you did not know before? |
| **Attitudes** | What do you think about having MyFood available at your ward, also in the future?   - Please explain why   Would you recommend other wards/hospitals to implement the use of MyFood?   - Why/why not?   What potential does MyFood or similar tools have in the follow-up of patients in Norwegian health care? |
| **Summary and closing** | Can you summarize the most important topics we have talked about in this conversation? |
