# Supplementary material for: Process evaluation of the implementation of a decision support system to prevent and treat disease-related malnutrition in a hospital setting
Source: BMC Health Serv Res. 2021 Mar 25;21:281. doi: 10.1186/s12913-021-06236-3 (PMC7995565; doi:10.1186/s12913-021-06236-3)
Supplement: Supplementary file 2 — Additional file 2: Supplementary file 2. Interview guide patients. [file 12913_2021_6236_MOESM2_ESM.docx]

**Process evaluation of the implementation of a decision support system to prevent and treat disease-related malnutrition in a hospital setting**

Mari Mohn Paulsen^1,2^, Cecilie Varsi^3^, Lene Frost Andersen^1^

**Author affiliations:**

^1^Department of Nutrition, Institute of Basic Medical Sciences, University of Oslo, box 1110 Blindern, 0317 Oslo, Norway.

^2^National Advisory Unit on Disease-related Undernutrition, Division of Cancer Medicine, Oslo University Hospital, Rikshospitalet, Oslo, Norway. Sognsvannsveien 20, 0372 Oslo, Norway.

^3^Center for Digital Health Research, Oslo University Hospital, Division of Medicine, Aker hospital, box 4959 Nydalen, 0424 Oslo, Norway.

**Corresponding author**: Mari Mohn Paulsen

[m.m.paulsen@medisin.uio.no](mailto:m.m.paulsen@medisin.uio.no)

**Supplementary file 2. Interview guide patients**

| **Introduction** | Purpose of the interview, practical information (audiotaping, confidentiality). |
| --- | --- |
| **Opening questions**  **The use of Myfood**  **Usability**  **Equipment and training**  **Experiences**  **Communication and culture**  **Perceived benefit** | What is your perception of the MyFood app?  What have you done in the MyFood app?  (Can you show me?)  What was good/useful?   - In what kind of way? - How could the app be more useful?   What is missing in the app?  Layout?  Use of pictures?  Meal categories?  How easy/difficult was it to use the app?  Did you find the food and beverages you consumed?  Did you find the meals you wanted to record?  Have you learned anything by using the MyFood app?  What kind of training did you receive beforehand?   - How did you experience the training?   What was your experience with using the MyFood app?   - Easy/difficult to remember to use the app? - Motivation?   How did you experience to report your food and drink intake?  Is there something you particularly liked in the app?  Is there something you think we should change in the app?  Whom did you talk to about the MyFood app?   - Healthcare personnel - Other patients - Next-of-kin - No one   How did the nurses use MyFood?   - Dialogue with you about your food intake? - Nutrition measures?   Do you have any thoughts about the use of the MyFood app compared with other nutritional follow-up?  Do you recommend others to use the MyFood app?   - Any patient groups/institutions MyFood is more suitable for than others? |
| **Participation in the study**  **Training/information** | How did you experience to participate in this study?  How did you experience the information you received?  How did you experience the training you received? |
| **Summary and closing** | Can you summarize the most important topics we have talked about in this conversation? |
